# Bringing down the Infantile Death-Rate

**Published:** 1917-12-01

**Authors:** 


### BRINGING DOWN THE INFANTILE DEATH-RATE.

### The Priestley Lecture of the National Health Society.

Sir Arthur Newsholme, K.C.B., speaking at the Robert Barnes Hall, 1 Wimpole Street, on November 21, on the "Enemies of Child Life, with special reference to Home Conditions," gave some most interesting figures proving to how large an extent infantile mortality is influenced by the dwelling and occupation of the mother during the months of pregnancy, and by the surroundings into which the child is born.

### The Proved Inaccuracy of a Common Statement.

The common statement that at birth all infants are equal in health is inaccurate, as is shown by (*a*) the differing incidence of stillbirths; (*b*) the differences in mortality rate of mothers after confinement, and (*c*) the widely varying statistics of infant mortality during the first week and the first months of life. Thus the stillbirths varied from 3 to 8 per cent. of the children born in different districts. In London the maternal mortality after child-bearing varied from 2.2 to 3.1 per 1,000 in various boroughs, whilst in Huddersfield and Oldham it was 6.1, in Dewsbury 6.5, and in Rochdale 7.2 per 1,000. No doubt the effect of malnutrition and over-work in bad surroundings, as well as alcohol and syphilis, are responsible to a great extent for these variations, as will be later shown in dealing with the lessened mortality under selected housing conditions. This is further emphasised by the deaths per 1,000 under one month of age. Workington heads the list with 61, Blyth and Batley next with 58, whilst Holborn and Reigate have but 28, Penge and Hornsey 27, and last and least Watford, with 26. During the first week, when environment can scarcely be said to enter into the question at all, thirty-four children die in the county of Durham, where women are employed to a large extent at the pit-head, to eighteen in suburban Leyton. Not only can we say that in Durham sixteen children, too many out of every 1,000, die in the first week of life, but that in Leyton under ideal conditions fewer children would die. The differences between urban and country mortality are, as might be expected, very great. In Oxfordshire the mortality is 63 per 1,000, in large towns the average is 99, and in the smaller towns 90. Thus the death-rate is 51 per cent. higher in large urban centres, and 34 per cent. higher in small towns than in county districts.

### Variations in the Mortality.

To how great an extent the bad effects of city life can be counteracted is shown in many ways. Thus in the same town there are wide and striking variations in child mortality in the different wards. In Middlesbrough two parts of the town show a mortality of 369 and 146 per 1,000 respectively, St. Helens 354 and 200, and even suburban Croydon 91 and 49. In districts regarded as specially favoured in this respect the figures also vary considerably. Thus Hampstead in some parts is as low as 69 and in others rises to 92, similarly Woolwich ranges between 64 and 110, Chelsea between 79 and 149, and, most striking of all, West Ham 79 and 171. That this is largely the direct result of insufficient and insanitary housing is demonstrated by the fact that the infantile death-rate in Peabody and other Trust buildings, where not only are the dwellings satisfactory from a hygienic point of view, but the tenants as a class are selected for their industry and sobriety, is in all cases less than that of the borough in which they are situated taken as a whole. In Finsbury, the mortality in the Trust buildings was 67 per 1,000, and the average for the borough 133, similarly in Stepney 74 and 122, and in Lambeth 83 and 104, whilst the average for the whole of London is 89 per 1,000. How potent an influence alcohol has upon the problem is strikingly illustrated by curves showing the *per capita* consumption of beer and spirits, the convictions per 10,000 population for drunkenness, and the infantile mortality rate. Over a long period of years these curves run almost parallel!

### Housing Requirements.

The urgency of the question has been enhanced during the last three years not only by the slaughter of tens of thousands of our young men upon the battlefields, but by reason of the enormous incursion of women into factories and workshops. Our birth-rate is steadily falling, and at the present rate the population will soon become stationary, as it already is in France. Limited families are now the rule and not the exception. More than ever the protection of the infant from adverse influences, ante-natal and environmental, is a matter of the first importance. The most needed reform, said Sir Arthur, was the insistence upon certain minimum structural requirements in the houses of the working classes. At present nearly three and a quarter million of the population, or 8.7 per cent., live in dwellings containing less than one room for every two persons, and four and a quarter million in three-roomed dwellings. Amongst other indispensables, a supply of gas, electric light, and water should be laid on at cost price, there should be separate lavatory and sink accommodation for every dwelling, and ample room for coal supply and food storage, whilst household refuse must be removed promptly and conveniently. Until these conditions are fulfilled it is foolish to talk of lower-class ignorance, and to say that "the tenant makes the slum." Health is always cheaper than disease, and within certain limits we can have as much health as we care to pay for.

### The Need of Mothercraft.

Sir James Crichton-Browne, the chairman, said that the German system of secondary marriages was a typical example of "Kultur," and would be abhorrent to English ideas. What was needed in this country was "mothercraft," and he had insisted that among the pamphlets distributed by health visitors there should be one entitled, "Precepts for Pregnancy." It has been estimated that after the war 300,000 new homes would be needed, and we must make it our business to see that every one of them was a real "home," where the child could receive a training that would last him throughout his life. Other speakers included Lady Rhondda, Sir Robert Morant, and Sir Ray Lankester.

